# Investigation of nasal epithelial cells as a surrogate for bronchial epithelial cells in the research of equine asthma

**DOI:** 10.1371/journal.pone.0293956

**Published:** 2023-11-09

**Authors:** Diane Frances Lee, David James Everest, William Cooley, Mark Andrew Chambers

**Affiliations:** 1 School of Veterinary Medicine, University of Surrey, Guildford, Surrey, United Kingdom; 2 Animal and Plant Health Agency, Addlestone, Surrey, United Kingdom; 3 School of Biosciences and Medicine, University of Surrey, Guildford, Surrey, United Kingdom; University of Pittsburgh, UNITED STATES

## Abstract

Equine asthma, previously known as Recurrent Airway Obstruction (RAO) or Inflammatory Airway Disease (IAD), is an often-debilitating condition that may severely affect both performance and quality of life. Research is hindered by the low sample numbers of subjects recruited to studies, a consequence in part of the invasive nature of the sampling methods of bronchial brushing and biopsy. We present an alternative method of sampling equine airway epithelial cells, the ‘nasal brush method’ (NBM). Obtained by light brushing of the ventral meatus whilst the horse is under standing sedation, these cells express the same markers of differentiation as their deeper counterparts. Grown as 3-D spheroids or as air-liquid interface cultures, nasal epithelial cells are responsive to the inflammatory cytokine interleukin-13. This may be attenuated by modulation of the Notch signalling pathway using the gamma-secretase inhibitor Semagecestat; a previously unreported finding that cements the link between equine and human asthma research and strengthens the case for a One Health approach in researching asthma pathophysiology and therapeutic intervention.

## Introduction

Equine asthma is the most common respiratory disease syndrome of the horse and is among the most common causes of training interruption and poor performance, particularly in the racing thoroughbred and endurance horse [1, 2]. The prevalence of asthma is high in racehorses (13–22%) [3] and sports horses (31%) [4], resulting in significant downtime of up to a third of the training period [5].

Meaningful quantities of equine bronchial epithelial cells (EBECs) for experimental studies of asthma can only be obtained *post mortem* [6, 7]. Alternatives, such as bronchial brushing and biopsy are invasive, requiring local anaesthesia in addition to sedation. Taking samples from healthy horses is therefore difficult to justify, is more costly and requires a recovery period of up to 48 hours [8]. This is why subject numbers in reported experimental studies are relatively low; typically less than 15 per group [9] with some as low as 2 [10]. This makes meaningful comparisons between healthy and diseased airways difficult and reduces statistical power, hampering progression towards more effective therapies.

Nasal brush sampling methods have been employed successfully for human research [11–14]; isolating basal epithelial cells that are cultured and differentiated into a heterogeneous epithelial population. In studies of respiratory disease, particularly cystic fibrosis [15, 16], conclusive evidence exists that nasal epithelial cells (NECs) may be used to study inflammatory airway diseases with the sampling procedure being well tolerated, even by infants. We therefore hypothesised that nasal brushing and isolation of equine NECs (ENECs) is likely to be a successful surrogate to bronchial brushing and biopsies when used to study asthma and other respiratory diseases of the racehorse. The less invasive nature of the procedure facilitates acquisition of samples, whilst re-sampling from the same animal is feasible and more justifiable from healthy donors, enabling greater study sample sizes and statistical power. Requiring only short-acting standing sedation and a procedure time of 15 seconds, downtime from training is negligible.

Significant attention has been given to 3-D spheroid cultures of airway epithelial cells (AECs) as an alternative to air-liquid interface (ALI) cultures [6, 7, 17] in the study of disease pathogenesis [18–20], owing to their ability to simulate tissue remodelling and regeneration. These have been used to demonstrate the major hallmarks of chronic inflammatory diseases of the human airway and the role of Notch signalling in the hypersecretory phenotype [21]. The differentiation of ciliated cells towards the secretory phenotype of the goblet cell via Notch signalling [22] and the knowledge that gamma-secretase cleaves the Notch intracellular domain (NICD) in the canonical pathway [23] has led to the suggestion of gamma-secretase inhibitors (GSIs) as alternatives to glucocorticoids and bronchodilators in the treatment of mild to moderate asthma [24, 25]. Demonstrated to attenuate the hypersecretory phenotype, including goblet cell metaplasia [23, 26], GSIs may prevent and reduce symptoms of horses in the early stages of asthma. To this end, we treated ALI and 3-D spheroid ENEC cultures with the inflammatory cytokine IL-13 to simulate a hypersecretory phenotype in the presence or absence of a GSI. Analysis of cell-specific markers of ciliated and secretory cells revealed an attenuation of IL-13 driven hypersecretory phenotype, supporting the use of ENECs as a surrogate for EBECs and in studies of disease pathogenesis pertaining to equine asthma. Comparison of healthy horses with those clinically diagnosed as asthmatic demonstrated significant differential expression of markers commonly associated with asthma, suggesting that the NBM may be developed further and used to complement current diagnostic methods in the clinic.

## Results

### Isolation of nasal epithelial cells offers a less invasive alternative to bronchial biopsy

**Fig 1:**
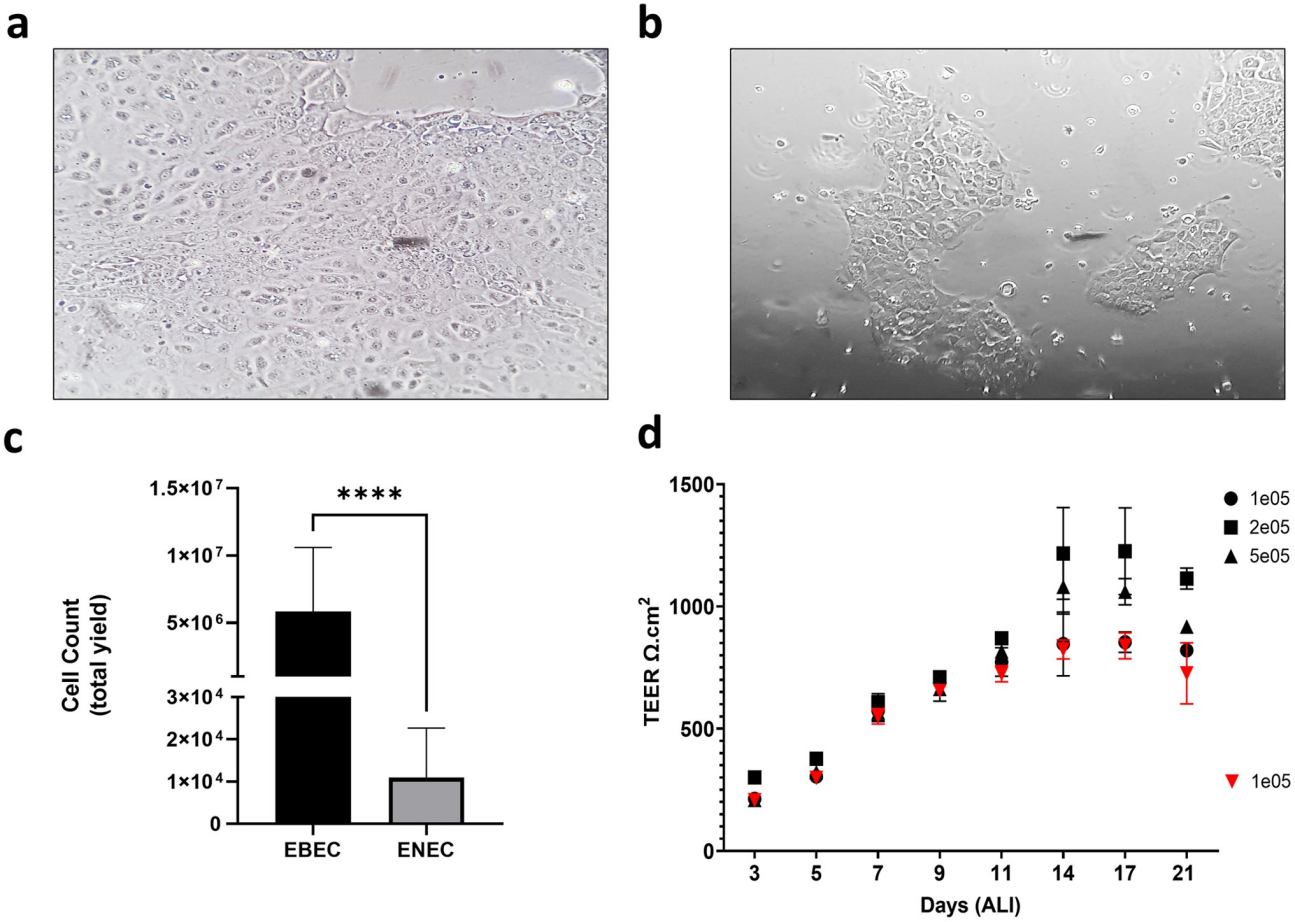
Characterisation of nasal brush isolated equine nasal epithelial cells (ENECs), compared directly with equine bronchial epithelial cells (EBECs). **a**) EBECs isolated via enzymatic digestion assemble in a cobblestone morphology characteristic of epithelial cells, generating a confluent monolayer. **b**) ENECs isolated using the nasal brush method form colonies with cobblestone morphology as per EBECs. **c**) Direct comparison of average yields between EBEC (5.82 ± 4.78 x 10^6^ cells per donor; n = 5) and ENEC isolations (1.10 ± 1.17 x 10^4^ cells per donor; n = 28) (p < 0.0001; Unpaired t-test). **d**) Trans-epithelial electrical resistance (TEER) measured across EBEC (black symbols) and ENEC air-liquid interface cultures (red symbols) seeded onto Greiner Thincerts. Measurements acquired from day 3 to 21 determined maximum TEER to be achieved by day 17 at all densities for EBEC. This finding was replicated in ENEC cultures, seeded at 1 x 10^5^ cells/cm^2^ only. Teer values between EBEC and ENEC cultures seeded at 1 x 10^5^ cells/cm^2^ showed no significant difference (p = 0.2994; Two-way ANOVA followed by Tukey’s Multiple Comparisons).

ENEC isolation was found to be a simple and effective technique to obtain airway epithelial cells from the horse. The method was well tolerated by standing short-term sedated horses and viable samples were consistently obtained. Only horses not showing clinical signs of asthma were sampled for the development of the NBM. At 24 hours post isolation, both EBECs (Fig 1a) and ENECs (Fig 1b) developed characteristic cobblestone morphology, however ENEC cultures were present as colonies, rather than an almost confluent monolayer. These colonies did not achieve 100% confluency when seeded as isolates from individual donors; most likely a reflection of total viable cell numbers obtained at the point of isolation, which differed significantly (unpaired t-test, P < 0.0001) between EBEC (5.82 ± 4.78 x 10^6^ cells per donor; n = 5) and ENEC (1.10 ± 1.17 x 10^4^ cells per donor; n = 28) (Fig 1c).

### Equine nasal epithelial cells are directly comparable with bronchial epithelial cells in 2-D and 3-D culture models

**Fig 2:**
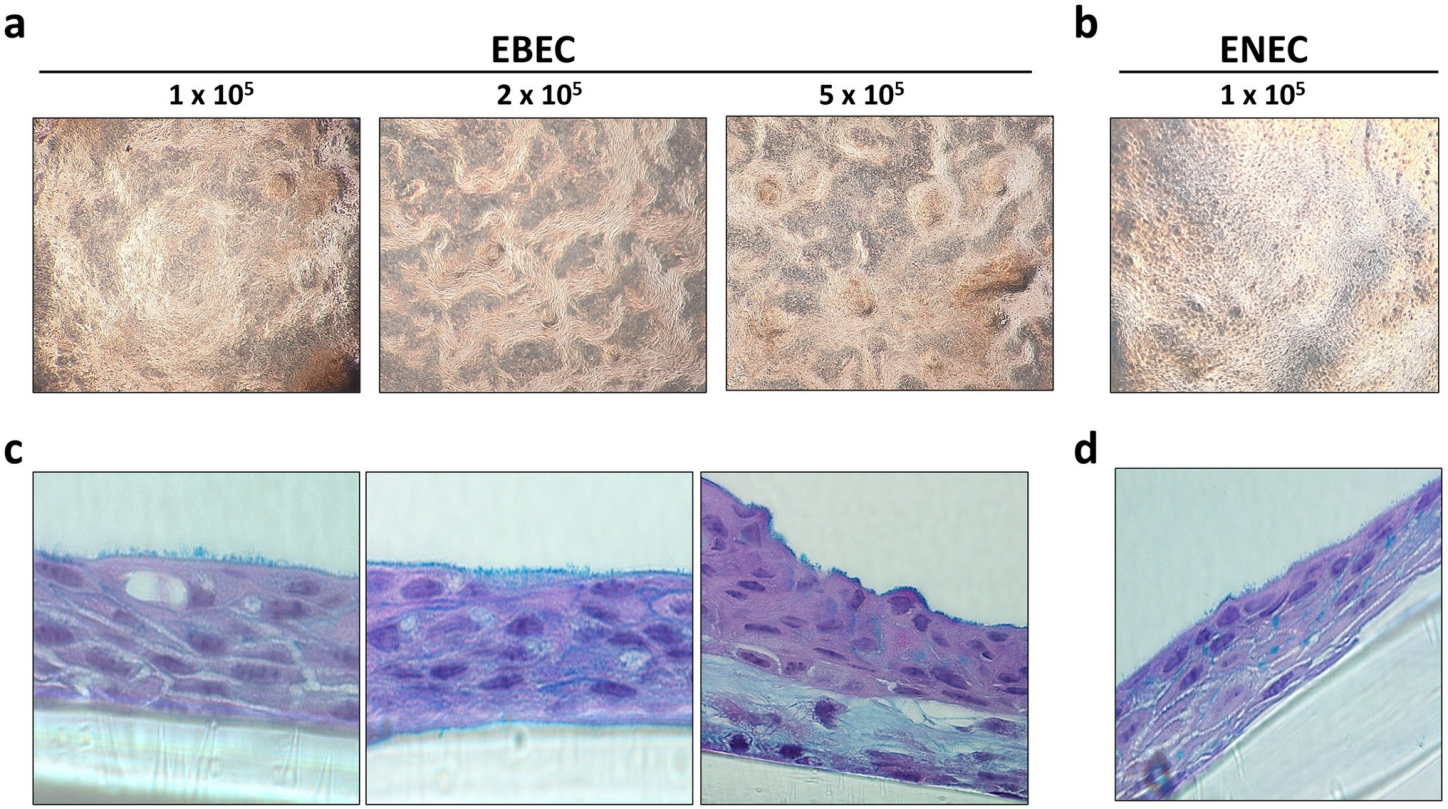
Direct comparison of morphology and histology between EBEC and ENEC cultures grown at air-liquid interface (ALI). EBECs were seeded at 1 x 10^5^ cells/cm^2^, 2 x 10^5^ cells/cm^2^ and 5 x 10^5^ cells/cm^2^ (**a**) onto 12 mm Greiner ThinCerts, 0.4 μm pore size. ENECs were seeded at 1 x 10^5^ cells/cm^2^ only (**b**). Each culture generated confluent layers of cells with classic ‘cobblestone’ appearance, with visible mucus on the apical surface (images acquired using 4 x objective). Corresponding haematoxylin and eosin staining of ALI cross sections, in which cilia can clearly be observed on the apical surface, at each seeding density and in both EBEC (**c**) and ENEC (**d**) cultures (images acquired using 100 x objective, with oil).

**Fig 3:**
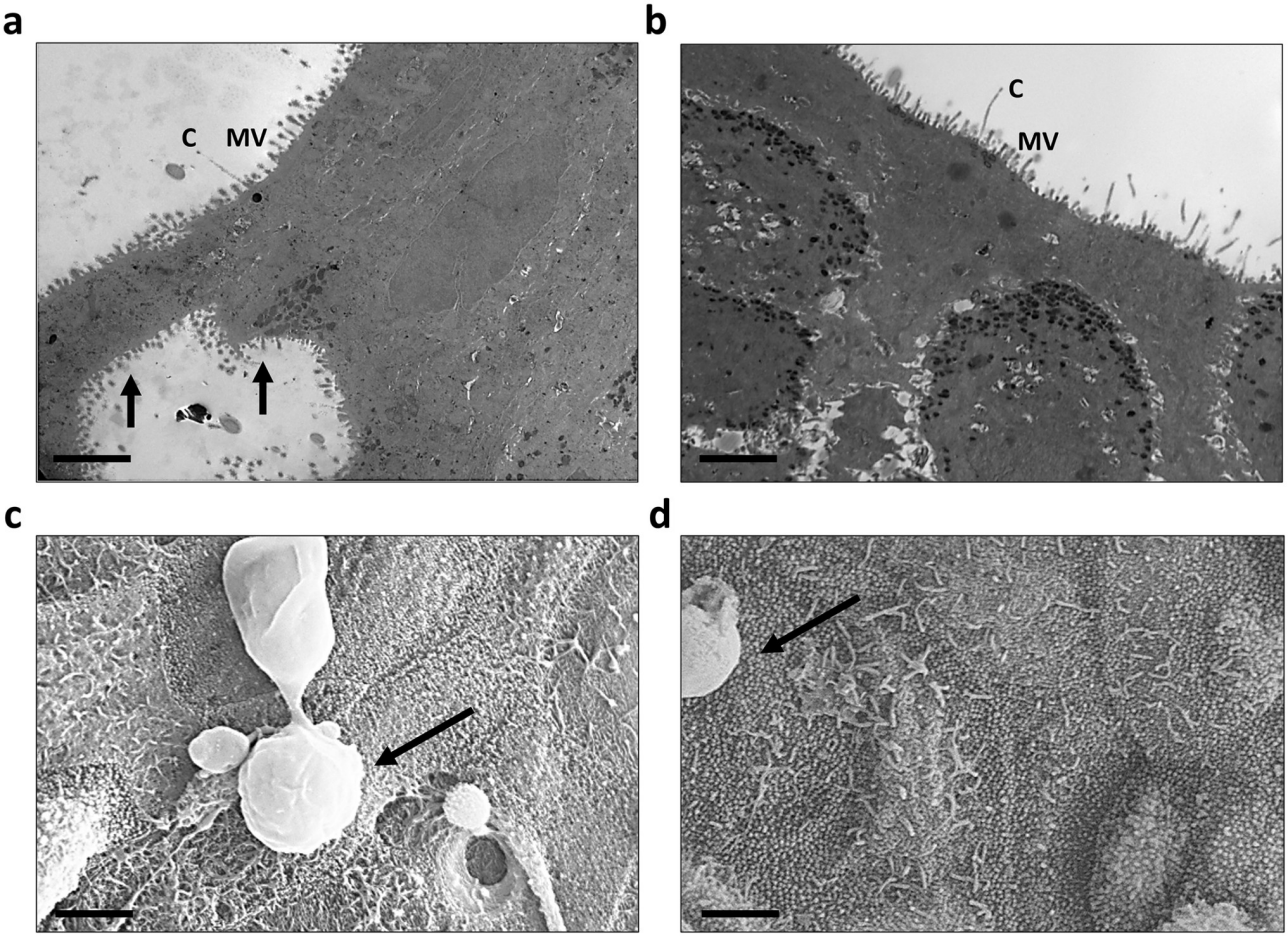
Transmission and scanning electron microscopy were used to confirm the presence of cilia-like structures on isolated bronchial and nasal epithelial cells. **a**) TEM revealed presence of long cilia-like (‘C’) structures present on the apical surface of EBECs cultured at ALI, together with shorter microvilli (‘MV’), also found in ‘lumens’ formed in layers of cells that had failed to form a pseudostratified epithelium (arrows). **b**) Cilia-like structures and microvilli were also observed in sections of ENEC cultures grown at ALI; TEM scale bars 2 μm. **c-d**) The observation of long cilia-like structures and microvilli in TEM images of EBEC (**c**) and ENEC (**d**) ALI cultures was replicated in SEM, in which mucus globules were also found (arrows); SEM scale bars 3 μm.

For morphometric analysis and assessment of differentiation potential, EBEC and ENEC cells were initially cultured at ALI. EBECs were seeded on 12 mm, 0.4 μm pore Greiner ThinCerts at three densities of 1 x 10^5^ cells/cm^2^, 2 x 10^5^ cells/cm^2^ and 5 x 10^5^ cells/cm^2^ to optimise culture conditions at ALI. ENEC isolates contained insufficient cell numbers as individual samples to seed at higher densities and were therefore only seeded onto ThinCerts at 1 x 10^5^ cells/cm^2^, by pooling two or more donors. Trans-epithelial electrical resistance (TEER) was measured from day 3 at ALI to day 21 (Fig 1d), with the finding that maximum TEER was achieved at day 17 for all seeding densities. EBECs recorded a maximum TEER of 854 ± 41.6 Ω.cm^2^ (1 x 10^5^ cells/cm^2^), 1226 ± 177.6 Ω.cm^2^ (2 x 10^5^ cells/cm^2^) and 1061 ± 54.1 Ω.cm^2^ (5 x 10^5^ cells/cm^2^). ENECs recorded a maximum TEER of 839 ± 53.2 Ω.cm^2^ (1 x 10^5^ cells/cm^2^ only). No discernible difference was observed in morphology between EBECs seeded at 1 x 10^5^ cells/cm^2^, 2 x 10^5^ cells/cm^2^ and 5 x 10^5^ cells/cm^2^ (Fig 2a and 2c). ENEC isolates were indistinguishable from EBEC cultures seeded at the same density of 1 x 10^5^ cells/cm^2^ (Fig 2b and 2d). The presence of long cilia-like and shorter microvilli structures was evident in both EBEC (Fig 2c) and ENEC (Fig 2d) cross-sections stained using the Alcian blue/Periodic acid-Schiff’s (AB/PAS) method, a finding that was confirmed by TEM (Fig 3a and 3b) and SEM (Fig 3c and 3d). Both EBEC and ENEC cultures failed to generate a consistently polarised pseudostratified epithelium, as evidenced by AB/PAS staining of cross sections. For this reason, we limited quantification of mucins to the ELLA and qPCR of mucin mRNA. What appeared to be hyperplasia was also observed in both cell types for the donors studied. This was not explored further due to issues with low yields generated by the NBM.

**Fig 4:**
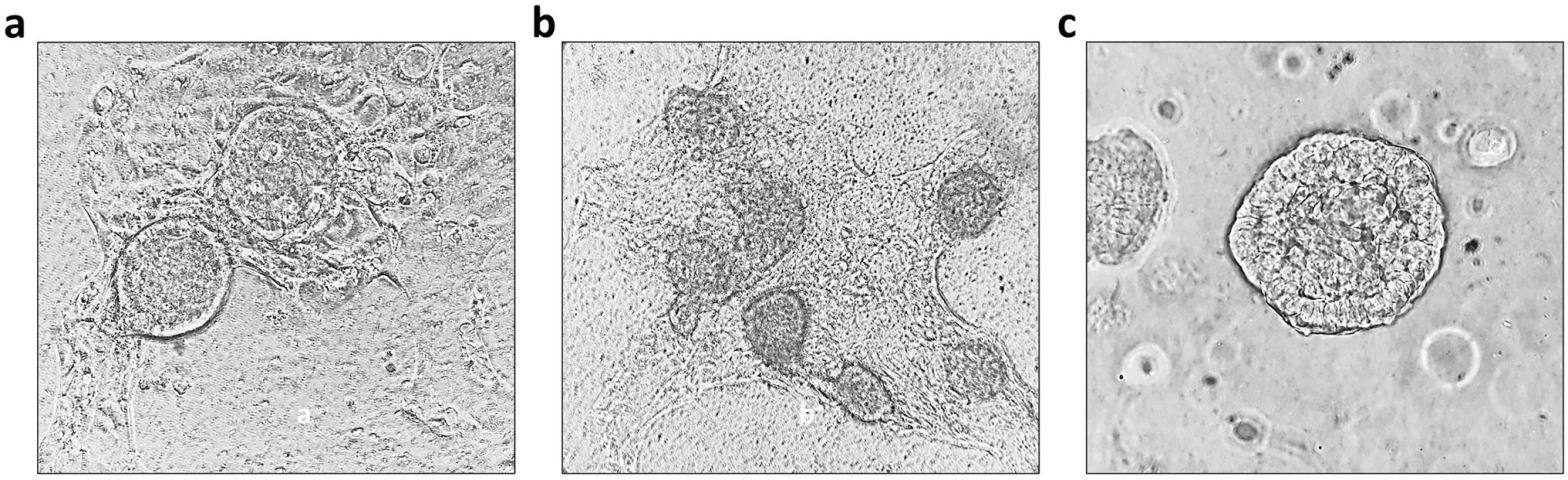
Morphometric analysis of EBEC and ENEC cells chambered coverglass cultures under the light microscope. **a**) EBEC and **b**) ENEC cultures formed 3-D spheroidal structures within 24 h of seeding, maturing into rounded organoids containing a central lumen (**c**) (example shown of ENEC spheroid). Images acquired using 40 x objective.

To address the ENEC low yield, cultures of both ENEC and EBEC at passage zero were studied by seeding cells directly into wells of a Nunc^™^ Lab-Tek^™^ II Chambered Coverglass slide pre-coated with PneumaCult-ALI (PC-ALI) medium containing 20% growth factor-reduced Matrigel. Both EBEC and ENEC formed 3-D spheroid-like structures (Fig 4a and 4b, respectively) within 24 h of seeding, maturing into rounded spheroids containing a central lumen (Fig 4c).

### IL-13 induces a mucus hypersecretory phenotype in EBECs and ENECs

**Fig 5:**
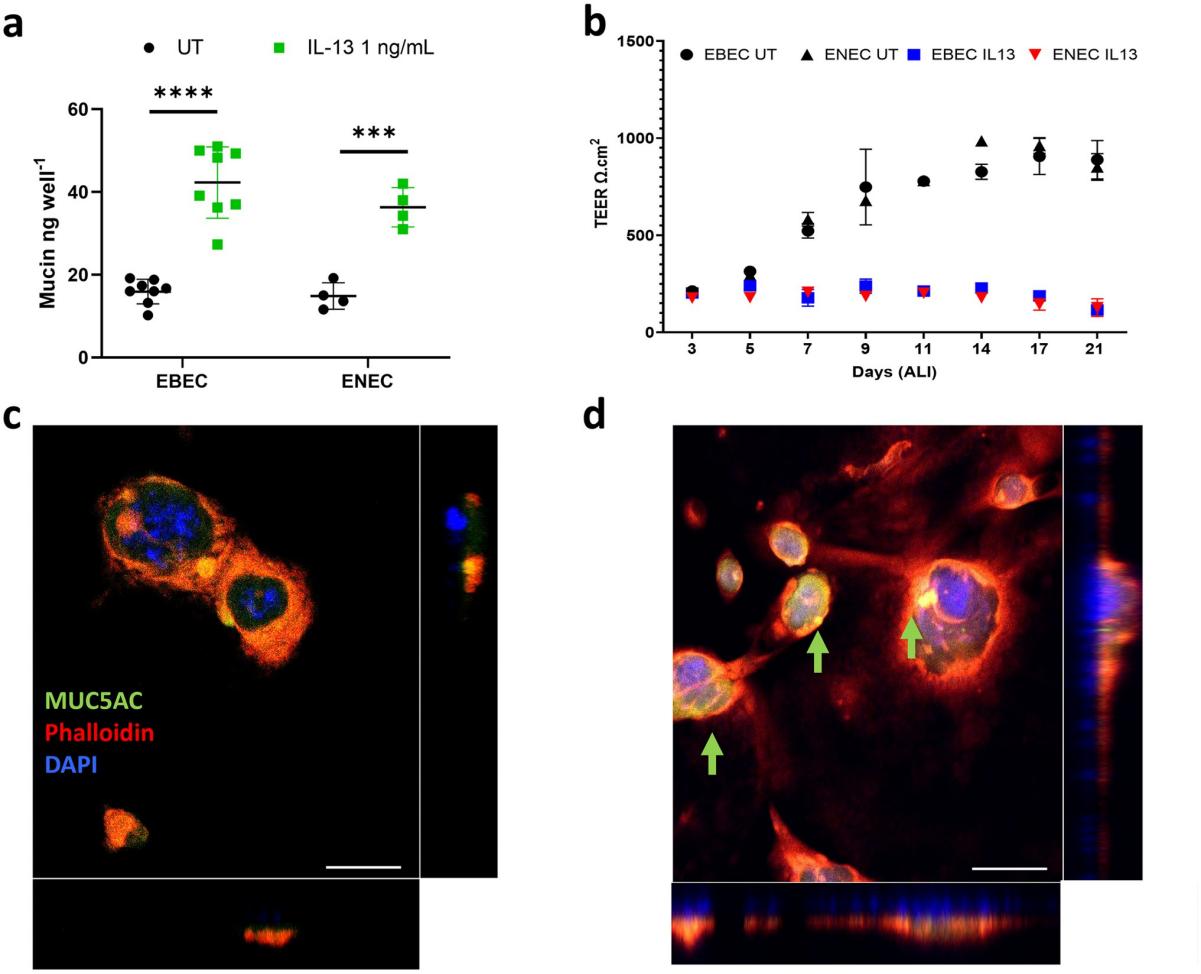
EBECs and ENECs adopt an asthmatic-like phenotype in response to treatment with the pro-inflammatory cytokine IL-13. **a**) EBEC and ENEC ALI cultures significantly upregulate secretion of mucins in response to 1 ng/mL IL-13 (Unpaired t-test). Data presented as Mean ± SD, n = 8 (EBEC) and n = 4 (ENEC). **b**) Increase in mucin secretion is mirrored by a drop in TEER to background levels (circa 100 Ω/cm^2^) when EBECs or ENECs are cultured at ALI in the presence of IL-13 1 ng/mL. Data presented as Mean ± SD, n = 3 for both cell types. **c-d**) ENEC spheroids stained with anti-MUC5AC appear to show an upregulation in MUC5AC when cultured in the presence of IL-13 1 ng/mL (green arrows denote intense areas of MUC5AC staining) (**d**). Scale bars 50 μm.

Previous studies have demonstrated the induction of a mucus hypersecretory phenotype using the pro-inflammatory cytokine IL-13 [21, 27, 28]; long since accepted as an inducer of mucin secretion in the respiratory epithelium [29, 30]. We therefore cultured EBEC and ENEC cells either at ALI or on chambered coverglass, in the presence of IL-13 (1 ng/mL), over 14 days, using a lectin-based assay to detect mucin as a function of mucous cell hyperplasia. Mucin content of apical surface washes increased from 15.95 ± 2.98 ng/well to 42.30 ± 8.62 ng/well (n = 8) in IL-13 treated ALI cultures of EBECs (Fig 5a). ENEC behaved similarly, with an increase in mucin content from 14.32 ± 0.99 ng/well to 40.01 ± 2.83 ng/well (n = 2) in ALI cultures (Fig 5a). Whilst a significant difference was observed between mucin content of untreated controls and IL-13 treated cultures for both cell types (Unpaired t-test; EBEC, P ≤ 0.0001; ENEC, P = 0.0003), no differences were observed comparing untreated EBEC with ENEC cultures or their IL-13 treated counterparts. Tight junction (TJ) integrity is commonly assessed by TEER [31]. Treatment with IL-13 has been shown to induce ubiquitination and proteasomal aggregation of TJ proteins, resulting in an increase in paracellular permeability and consequently, a decrease in TEER [32]. Here, TEER measurement decreased significantly for both EBEC and ENEC ALI cultures treated with IL-13, with no significant difference of TEER being observed between untreated EBEC and ENEC cultures (Unpaired t-test, P = 0.8970) or their IL-13 treated counterparts (Unpaired t-test, P = 0.1672) (Fig 5b). In agreement with these findings, staining of ENEC spheroid cultures with anti-MUC5AC antibody appeared to show an upregulation of MUC5AC protein expression in response to IL-13 treatment, however MUC5AC signal was not quantified in these images, due to the 3-dimensional nature (Fig 5c and 5d).

### The IL-13 driven mucus hypersecretory phenotype is attenuated by treatment with the GSI Semagecestat

**Fig 6:**
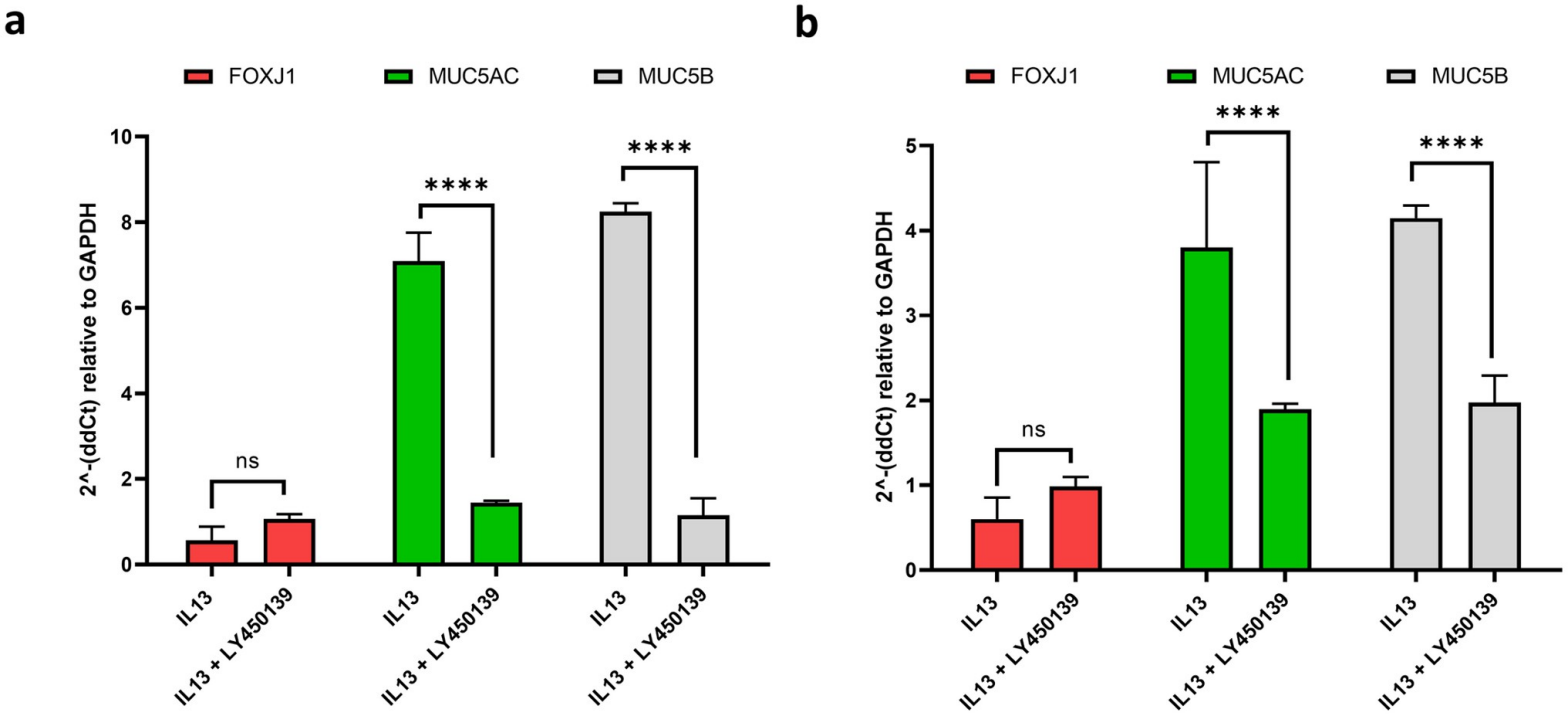
Expression of secretory cell and ciliated cell markers by EBEC and ENEC cultures is altered by inclusion of IL-13 (1 ng/mL) in culture media. This is attenuated by addition of the GSI Semagecestat. **a**) Treatment of EBEC cells with IL-13 induced upregulation in *MUC5AC* (7-fold, p < 0.0001) and *MUC5B* (8-fold, p < 0.0001) mRNA expression, whilst expression of the ciliated marker *FOXJ1* was downregulated (0.6-fold, n.s at p = 0.4987)(Two-way ANOVA, followed by Tukey’s Multiple Comparisons). Inclusion of Semagecestat in the culture medium, attenuated the increase of *MUC5AC* mRNA (to 1.4-fold), whilst restoring both *FOXJ1* and *MUC5B* to untreated levels (1.1-fold change). **b**) Attenuation of the IL-13 induced asthmatic phenotype was replicated in ENEC cultures. A 4-fold change in *MUC5AC* was reduced to 2-fold and *MUC5B* reduced from 4-fold to 2-fold. Expression of *FOXJ1* was again reduced in the presence of IL-13 to 0.6-fold, returning to untreated levels (1-fold) in the presence of Semagecestat. Data presented as Mean fold change ± SD; n = 2.

The Notch signalling pathway regulates cellular developmental fate decisions [22, 33, 34]. Selective blockade of individual Notch ligands either directly (antibody [21]) or indirectly through inhibition of gamma-secretase mediated cleavage of the notch intracellular domain (NICD) has led to the proposal of manipulation of the Notch signalling axis as a therapeutic strategy in the treatment of chronic inflammatory diseases, including asthma [35]. In the current study, IL-13 (1 ng/mL) induced upregulation of *MUC5AC* (7-fold) and *MUC5B* (8-fold) mRNA expression in ALI cultured EBEC (Fig 6a) and ENECs (Fig 6b) was attenuated in cultures additionally treated with Semagecestat (1 μM) over the 14-day ALI period, down to a 1.4-fold and 1.1-fold increase in mRNA expression, respectively. In contrast, the ciliated marker *FOXJ1*, downregulated to 0.6-fold (of controls) in cultures treated with IL-13, was restored to untreated control levels in cultures additionally treated with Semagecestat.

**Fig 7:**
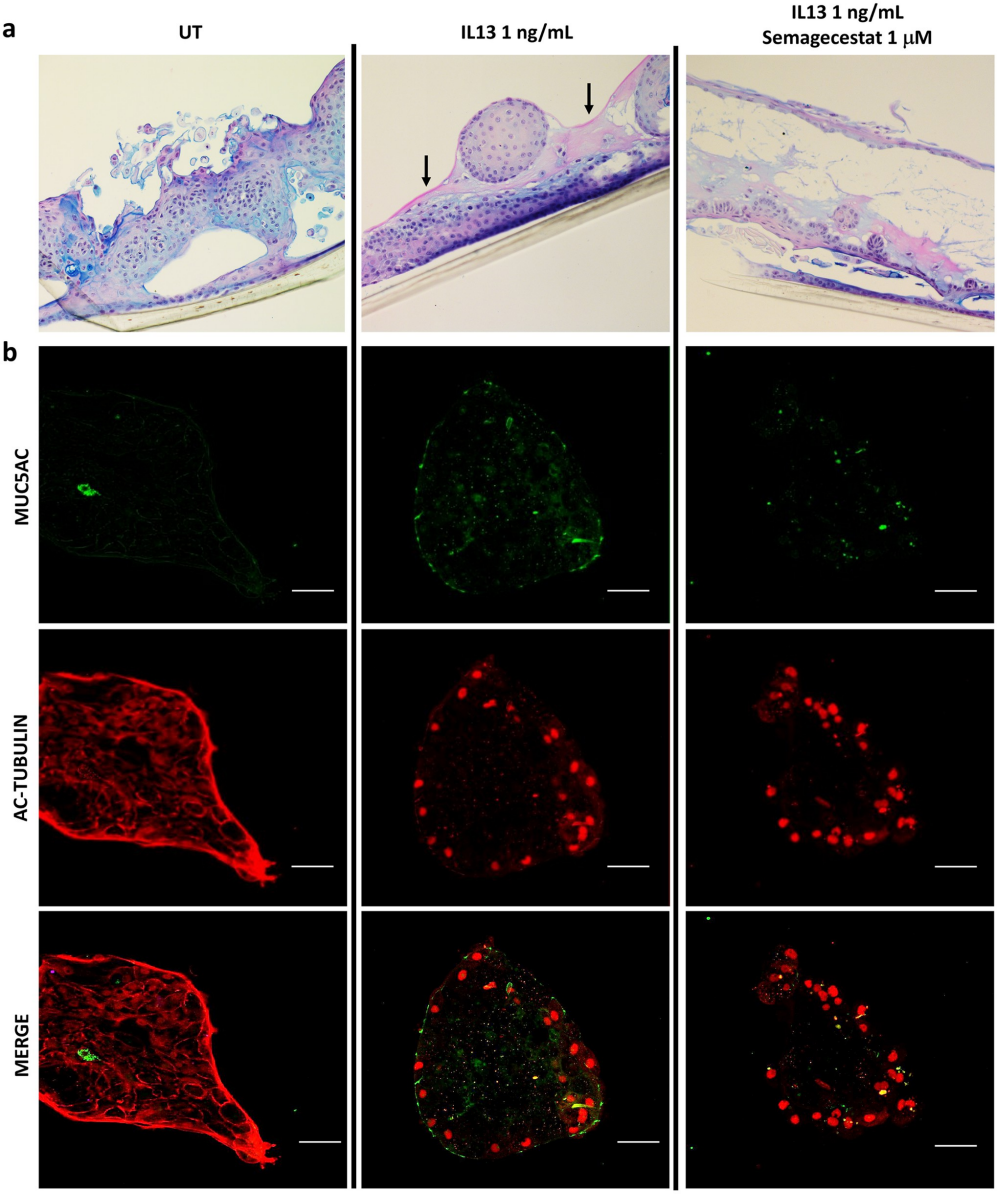
Addition of the GSI Semagecestat to IL-13 treated cultures of ENECs reduces mucin secretion as detected by periodic acid-Schiff’s (PAS) stain and immunofluorescence. **a**) Neutral mucins are stained magenta by PAS, whilst DNA/nuclei are stained blue-purple by haematoxylin. PAS appears to be intensified in cultures treated with IL-13 1 ng/mL (middle panel, arrows), with staining apparently reduced to control levels in cultures treated simultaneously with Semagecestat. Images acquired using 20 x objective and are representative of a minimum of 3 sections. **b**) Spheroid cultures treated with IL-13 only (1 ng/mL) exhibited stronger signal for MUC5AC (green) and an altered pattern for acetylated-tubulin (red). The increase in MUC5AC signal effected by IL-13 was lessened in the presence of Semagecestat. Scale bars 50 μm.

To ascertain whether this difference in mRNA expression translated to the phenotype, ENEC ALI culture cross sections were stained with AB/PAS reagents, commonly used to visualise mucins. Despite failing to form polarised layers at ALI, ENECs cultured in the presence of IL-13 (1 ng/mL) stained strongly with PAS (magenta) on the apical surface, indicative of increased mucin secretion (Fig 7a, middle panel, arrows). By eye, staining intensity was reduced when Semagecestat 1 μM was also included in the culture medium (Fig 7a, right hand panel). Spheroid cultures grown on chambered coverglass slides revealed a similar increase in mucin in response to IL-13 when probed with anti-MUC5AC (Fig 7b, middle panel), again displaying a partial reversal to the control phenotype when cultured concurrently with Semagecestat in the media (Fig 7b, right hand panel). As well as a change in MUC5AC expression, a change in the distribution of acetylated-tubulin in response to IL-13 treatment was observed, from cytoplasmic (UT) to predominantly nuclear. This was unaffected by addition of Semagecestat at 1μM.

### Nasal epithelial cells show potential as an additional diagnostic tool for equine asthma

Definitive diagnosis of equine asthma is obtained through a combination of airway endoscopy and characterisation of bronchoalveolar lavage (BAL) fluid cytology. The lack of tests and biomarkers available limits the ability to accurately diagnose in the field [36], since endoscopies and BAL may only be performed in a clinical setting. Diagnosis thus remains a challenge in the field due to comorbidities with other respiratory and non-respiratory diseases [37–39]. In the current study, mRNA was isolated from ENECs taken from healthy donors and horses previously diagnosed by their primary practice with moderate to severe asthma (summary of diagnostic parameters in Table 1). Nasal brush sampling was performed during referral lameness diagnostics or dental procedures undertaken by Bell Equine Clinic [40]. Asthma was previously diagnosed by the primary (referring) practice by virtue of the presence of outwardly visible symptoms (flared nostrils, elevated respiratory rate and presence of cough in absence of infectious disease), endoscopy to identify excessive mucus and bronchoalveolar lavage fluid (BALF) cytology (BALF performed on 5 out of 7 horses included in the study).

**Table 1:** 

	Healthy	Asthma
Number of horses	11	5^a^
Mean ± SD age, years	8 ± 4.79	9 ± 3.45
Sex m/g/s	5/4/2	2/5/0
Mean ± SD resting respiratory rate	18 ± 0.98	20 ± 1.21
Mean total cell count	N/A^b^	444.20 ± 81.98
Neutrophils, mean ± SD %	N/A	19.86 ± 6.40
Mast cells, mean ± SD %	N/A	2.02 ± 0.29

Summarised data containing details of horses sampled using the nasal brush method for the purposes of mRNA extraction and qRT-PCR analysis

^a^2 horses were not assessed for cytology by the diagnosing primary clinic and were diagnosed on symptoms alone

^b^N/A—not available

**Fig 8:**
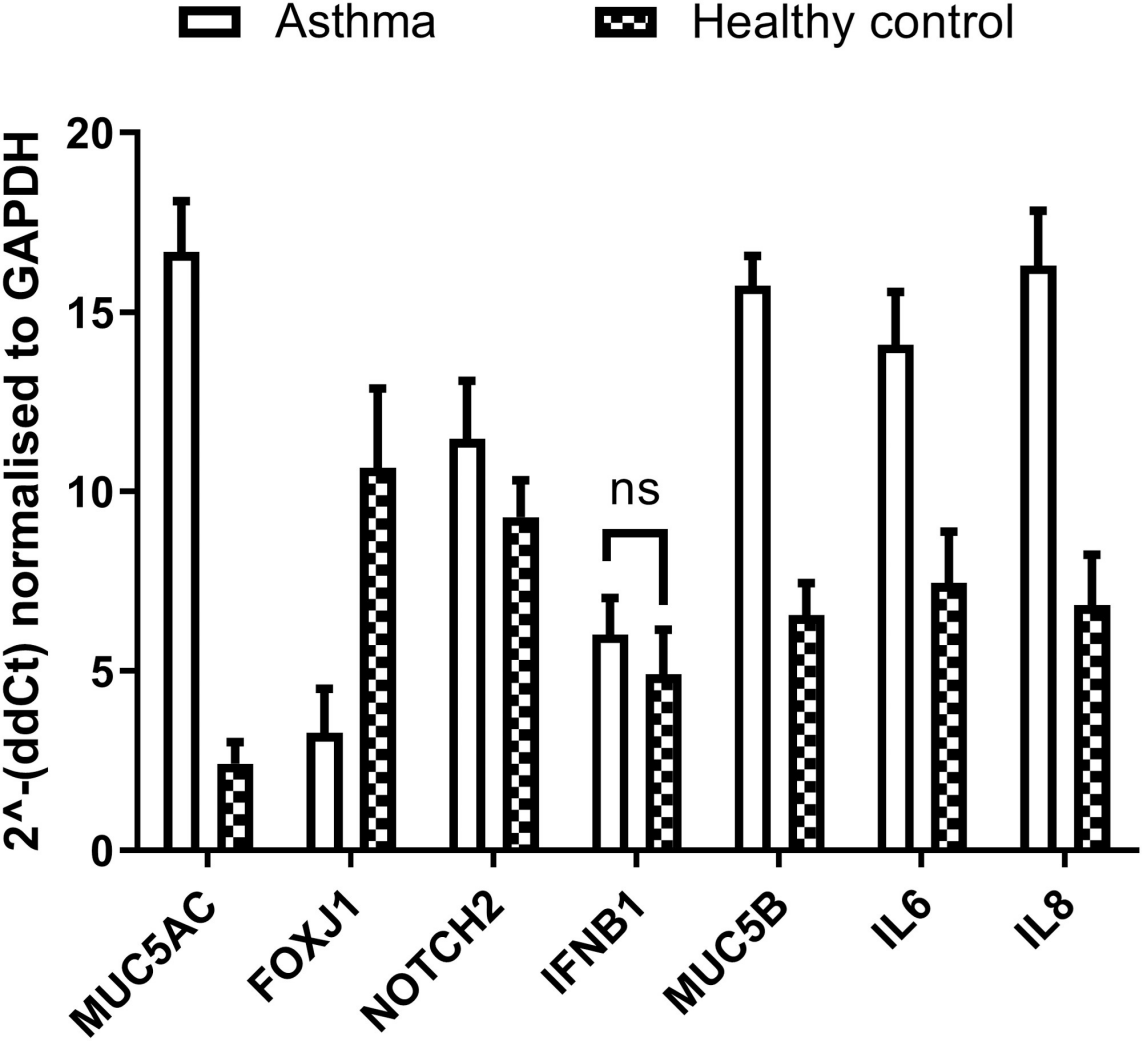
The nasal brush method may form the basis of an additional diagnostic strategy for equine asthma. Comparing mRNA isolated from ENECs between healthy donors and horses diagnosed with moderate to severe asthma, differential expression was observed in *MUC5AC* (p < 0.000001), *FOXJ1* (p < 0.000001), *NOTCH2* (p = 0.003), *MUC5B* (p < 0.000001), *IL-6* (p < 0.000001) and *IL-8* (p = 0.000009) (multiple Unpaired t-tests). Data presented as Mean ± SD, n = 7 (asthma)/ n = 11 (healthy control).

Isolated mRNA was subjected to reverse transcription and the resulting cDNA studied using qPCR of genes previously reported to be differentially expressed in asthmatic (human) subjects. Threshold cycle (Ct) levels of *MUC5AC*, *FOXJ1*, *NOTCH2*, *IFNB1*, *MUC5B*, *IL-6* and *IL-8* were normalised to GAPDH and compared between the two groups, relative to equine total lung RNA. When comparing healthy controls with asthmatic subjects (Fig 8), differential expression was observed in *MUC5AC* (p < 0.000001), *FOXJ1* (p < 0.000001), *NOTCH2* (p = 0.003), *MUC5B* (p < 0.000001), *IL-6* (p < 0.000001) and *IL-8* (p = 0.000009) (multiple Unpaired t-tests), suggesting that these markers may be used in the diagnosis of equine asthma using the nasal brush technique. There was no significant difference in the expression *IFNB1* between healthy control and asthmatic subjects.

## Discussion

Asthma is one of the leading causes of poor performance, debilitation and a reduced quality of life for horses [2, 39]. Live animal studies of the disease may be considered unethical, whilst *in vitro/ex vivo* studies are hindered by technical difficulties. Acquiring EBECs from live animals necessitates heavy sedation, local anaesthetic and by consequence a recovery period [8] unless achieved through licensed horse slaughter facilities, however this is fraught with challenges of logistics and availability [41].

This study investigated the feasibility of using ENECs as a surrogate for EBECs using a nasal brushing technique, the ‘nasal brush model’ (NBM). This technique was well tolerated by horses under short-acting standing sedation. Whilst the thoroughbred was the preferred breed for this work, COVID19 pandemic restrictions necessitated removal of breed and age criteria to enable sufficient samples to be collected. A total of 31 healthy horses were sampled using the NBM, of which 28 were viable. Cell yield made ALI culture challenging, therefore ALI experiments were limited to essential characterisation (TEER measurements, morphology, and mucin quantification). Several equine asthma studies report successful culture of EBECs at ALI [6, 7, 42, 43], however the limitations of ENEC ALI culture could not be overcome without pooling samples. Outside pandemic limitations, this is a feasible option and would reduce donor variability, providing that pooled cultures are well characterised with multiple replications [44], although it reduces statistical power [45]. To address low yields and subject numbers, we cultured EBECs and ENECs as 3-D spheroids, as studied in human models [21, 46, 47]. For these reasons, the current study did not explore further why EBEC and ENEC cultures did not form pseudostratified epithelia at ALI; although the authors intend to conduct further optimisation to expand use of the model to infection studies, drug permeability and toxicity, including but not exclusive to different culture media (with separate species-specific growth factors and horse serum supplementation). Potential reasons for this failure include an inappropriate substratum or subclinical disease of the donor, resulting in basal cell hyperplasia as observed in human COPD [48]. It is possible that the donors of EBECs used in the current study were compromised by disease undiagnosable by gross tissue inspection.

The attenuation of the mucus hypersecretory phenotype in ENECs builds upon previous studies performed with human airway epithelial cells, whereby gamma-secretase inhibition was identified as a potential strategy for reducing secretory cell differentiation. Glucocorticoid (GC) use in horses remains controversial, partly as their use is based upon extrapolation from human studies [49], but also due to the potential of GCs to be performance enhancers, giving rise to their prohibition in competition [50]. Furthermore, GCs are of limited efficacy against cytokine (IL-13) mediated mucus hypersecretions [51]. Acting on the canonical Notch signalling pathway by inhibiting cleavage of the NICD [23], GSIs have gained favour as an alternative to GCs in the treatment of human asthma [52], so it is possible that GSIs may improve symptoms of equine asthma. Addition of a GSI to IL-13 treated ENEC cultures resulted in attenuation of the hypersecretory phenotype by mRNA and protein expression. It was our intention to use acetylated tubulin as a marker of cilia in this study and therefore the change in distribution was unexpected. Altered distribution of acetylated-tubulin in response to IL-13 treatment has been reported previously in numerous cancer cell lines and other highly proliferative cells [53]. Predominantly found in structures containing long-lived stable microtubules (cilia, flagella and centrioles), abnormal acetylation has also been reported in neurodegenerative disorders. In Parkinson’s, for example, aberrant microtubule structures such as perinuclear cages are formed, mediated by tubulin polymerisation-promoting protein/p25 (TPPP/p25) [54]. We therefore attribute our findings to the maintained presence of IL-13 in culture media, inducing cellular stress and consequentially, aberrant acetylation of tubulin.

Finally, we explored the possibility of assembling a qRT-PCR panel of differentially expressed genes in asthma. Over-diagnosis of equine asthma results in unnecessary treatment and a delay in making a correct diagnosis, whilst under-diagnosis risks serious exacerbations and lung remodelling, an extrapolation from human medicine [55]. The lack of cell count data from some asthmatic horses in the current study highlights the need for better tests and biomarkers for which sample may be obtained in the field. Our preliminary findings were that a selection of genes commonly associated with asthma were consistently differentially expressed in asthmatic equine patients relative to total equine lung RNA, compared to healthy controls. As the sampling of both healthy and asthmatic horses here was independent of clinical investigations into respiratory disease, it was outside the study’s ethical considerations and approval to perform BAL on either group, so further developments into the use of qPCR as a diagnostic tool should include BAL in healthy controls and the resulting data on total cell count, neutrophils and mast cells compared between healthy controls and asthmatic horses accordingly. If successful, the NBM and subsequent mRNA extraction of samples would offer a viable and easily implemented alternative to BALF characterisation and endoscopy.

In conclusion, we present here a viable method of ENEC acquisition and propose ENECs as a more readily available and suitable surrogate for EBECs. The NBM shows potential for use in future studies exploring the use of human therapies in the horse (and reverse), conducive to the One Health ethos, expediting achievement of the goals of good asthma control, minimisation of symptom burden and risk of exacerbations.

## Methods

### Isolation and culture of equine bronchial epithelial cells (EBECs)

Tissue was excised from the tracheal bifurcation of horses of unknown age euthanised by a local fallen stock facility. Reasons for euthanasia were not disclosed other than a declaration that they were not related to respiratory disease. The tissue was transported in a sealed vessel containing Dulbecco’s phosphate buffered saline (DPBS) (Thermo Fisher Scientific, Waltham, MA) and Primocin (100 μg/mL) (Invivogen, San Diego, CA) to the laboratory. The respiratory mucosal surface (total of ~40 mL by volume) was washed with fresh DPBS/Primocin, before stripping mucosa from the submucosa using sterile forceps and scalpel. These were dissected into pieces approximately 3 mm^2^ in size. These were washed a minimum of four times (or until no red discolouration—indicating blood contamination—was observed) in DPBS/Primocin by vigorous shaking. The pieces were then added to 50 mL tubes for enzymatic digestion; splitting to ensure pieces did not occupy > 10 mL in volume per tube. Digestion solution (30 mL, consisting of Trypsin 0.25%/ ethylene diamine tetra acetic acid sodium salt (EDTA) 1 mM (Thermo Fisher Scientific) / Primocin 100 μg/mL) was added to each tube before incubation on a roller at 37°C for a total of 4 h. The pieces were then strained from each tube by serial filtration through cell strainers (Corning, New York, NY) of 100 μm, 70 μm and 40 μm mesh sizing. Strained cells in suspension were spun at 300 x *g*, washed in DPBS/Primocin 100 μg/mL twice and resuspended in complete PneumaCult Ex (PC-EX) (StemCell Technologies, Vancouver, Canada) supplemented with PneumaCult-Ex 50x supplement and hydrocortisone 200 x supplement (both StemCell Technologies), containing Primocin 100 μg/mL for counting and viability determination on the BioRAD TC20 cell counter. StemCell Technologies did not disclose the formulation of the 50x supplement supplied with this medium. Cells were seeded onto Collagen I (ready to use solution, Thermo Fisher Scientific) coated plastic at a density of 1x 10^5^ cells/cm^2^. Cells were routinely passaged at 80% confluence by a 1 in 2 split, by rinsing with DPBS and incubating for 5 minutes with 0.25% Trypsin/1 mM EDTA. Frozen stocks were prepared by pelleting first to third passage cells (1 x 10^6^ cells per cryovial) and resuspending in 1 mL Cell Recovery solution (Thermo Fisher Scientific) for storage in liquid nitrogen.

### Isolation of equine nasal epithelial cells (ENECs)

Horses of known age and breed visiting the clinic for ongoing lameness investigation or dentistry were recruited at the Bell Equine Clinic, Mereworth, UK, following completion of an Owner Consent Form. No exclusions were placed with regards to age or breed, but horses undergoing asthma treatment, diagnosed with bacterial, viral, or fungal infection or with a known history of respiratory disease were excluded. Horses were sedated for the booked investigation procedure (performed under the Veterinary Surgeon’s Act (1966)) and the procedure carried out. Whilst under short-acting standing sedation, a cotton swab dipped in sterile DPBS was used to wipe excess mucus from the ventral meatus of each nostril. A Cytotak^™^ Transwab^®^ cervical brush (Medical Wire and Equipment Co Ltd, Corsham, UK) was then inserted and rubbed gently up and down the ventral meatus surface, rotating the brush for 15 seconds before retraction and placement of the brush into a sample tube containing ice-cold DPBS/ Primocin 100 μg/mL. Tubes were placed on ice for transport to the laboratory, where the tube was gently vortexed to dislodge cells from the brush. The suspension was transferred to a 15 mL conical tube, spun at 200 x *g* and the supernatant discarded. Fresh DPBS/Primocin 100 μg/mL was used to repeat the wash step a further two times before cells were resuspended in 0.5 mL PC-EX for counting. Cells were seeded into Collagen I coated 24 well plates at a density of 1 x 10^5^ cells/cm^2^, culturing as per EBECs.

### Culture of EBECs and ENECs at air-liquid interface and as spheroids

For ALI culture, cells were seeded at first passage at a density of 1, 2 or 5 x 10^5^ cells/cm^2^ onto uncoated 12 mm Greiner ThinCert cell culture inserts containing transparent permeable polyester (PET) membranes, 0.4 μm pore size (Greiner Bio-One, Kremsmünster, Austria). Cells were maintained for 7 days by feeding apically (0.5 mL) and basolaterally (1.5 mL) with PC-EX containing Primocin 100 μg/mL. At 7 days, the medium was switched to PneumaCult ALI (PC-ALI) (StemCell Technologies) supplemented with PneumaCult-ALI 50x supplement, PneumaCult-ALI 10x Maintenance supplement and heparin sodium salt at 2.0 U/mL (all StemCell Technologies), feeding from the basolateral aspect only.

Alternatively, for spheroid culture, cells were seeded at a density of 4 x 10^3^ cells in 200 μL PC-ALI containing 5% growth factor-reduced Matrigel (Corning) and Primocin 100 μg/mL, into wells of a Nunc^™^ Lab-Tek^™^ II Chambered Coverglass slide (Thermo fisher Scientific), pre-coated for 1 hour at 37°C with PC-ALI (100 μL per well) containing 20% growth factor-reduced Matrigel. Each well was fed on day 3 post seeding and day 8, adding 200 μL PC-ALI containing 5% growth factor-reduced Matrigel following careful removal of the uppermost 200 μL of each well. Harvest or fixation for immunofluorescence and histology was performed on day 14 of ALI or spheroid culture.

### Mucin detection by lectin assay

Cells cultured at ALI were washed with 150 μL of DPBS. A sample (100 μL) was added to a well of a 96-well clear bottomed, white walled assay plate in duplicate, alongside serial dilutions of porcine gastric mucin (Merck & Co., Inc., Kenilworth, NJ) to generate a standard curve of 50, 25, 12.5, 6.25, 3.125, 1.56, 0.78 and 0.39 ng/well. Mucin was allowed to bind overnight at 4°C, then each well was washed three times with DPBS/1.0% gelatin/0.05% Tween 20 (Wash Buffer, WB) (all Merck). The plate was blocked with 150 μL PBS/1.0% gelatin for 1 h at 37°C, then washed three times with 200 μL WB. Lectin from *Triticum vulgaris* conjugated to fluorescein isothiocyanate (FITC, 100 μL, 5.0 μg/mL) was added to all wells and the plate returned to 37°C for 1 h. All wells were washed as before, then the plate was read on a ClarioStar Plus plate reader (BMG Labtech, Ortenberg, Germany) with an excitation wavelength 485 nm and emission wavelength 520 nm. Unknowns were interpolated from the standard curve using GraphPad Prism v9.0.0 (GraphPad Software Inc., San Diego, CA).

### Treatment of cultures with IL-13 and/or the GSI Semagecestat

Where cultures were treated with IL-13 (1 ng/mL), or IL-13 combined with Semagecestat (2 nM) (Merck), this was added to the feeding medium on the appropriate day. Treatments were added to Greiner ThinCert cultures from Day 1 of ALI or from day of seeding for spheroid cultures.

### Immunohistochemistry

Whole inserts were fixed in 10% neutral buffered formalin (NBF) (Merck) and were dehydrated sequentially in 70, 90 and 100% ethanol, for 30 min at each concentration. They were then incubated in 100% isopropanol for 30 min, transferred to molten paraffin wax (65 °C) for 1 h and the membrane teased from the insert using a scalpel blade. The membrane was embedded in paraffin, cured, and sectioned at 4 μm. For haematoxylin and eosin (H&E) staining, sections were dewaxed and stained as outlined previously [56]. Stained sections were dehydrated and mounted in DPX mounting medium (Merck) with a cover slip overlaid for analysis on a Nikon Eclipse Ci upright microscope. Alcian blue and periodic acid-Schiff’s (AB-PAS) staining was performed on rehydrated sections using an Alcian Blue PAS Stain Kit (Abcam, Cambridge, UK) according to the manufacturer’s protocol. Dehydration and imaging of stained sections was performed as per H&E.

### Immunofluorescence

The primary antibodies used in these studies were MUC5AC (clone 45M1;Thermo Fisher Scientific), FOXJ1 (HPA005714; Merck), acetylated tubulin (clone 6-11B-1; Merck) and zonula occludens (INV 40–2200; Thermo Fisher Scientific). Secondary antibodies Alexa 488, Alexa Fluor 568, rhodamine-conjugated phalloidin, and ProLong gold antifade mountant were all obtained from Invitrogen. NucBlue nuclear stain was obtained from Thermo Fisher Scientific.

Slides were washed gently with DPBS and fixed in 10% NBF at room temperature for 15 minutes, and permeabilised by washing in DPBS containing 0.1% Triton X-100 (Merck) for 15 minutes. Blocking was performed for 1 hour at room temperature in DPBS/ 5% normal goat serum (Merck) / 0.1% Triton X-100. Primary antibodies were diluted 1/100 in blocking buffer, applied to cells and incubated overnight at 4°C. Cells were rinsed three times with DPBS and secondary antibody applied for 1 hour at room temperature in the dark. Cells were again rinsed three times with DPBS before adding rhodamine-conjugated phalloidin according to manufacturer’s instructions (diluted in DPBS), and/or with 2 drops NucBlue DNA stain per mL. This was removed and replaced with 200 μL DPBS for imaging on a Nikon Eclipse Ti confocal microscope.

### Measurement of cell layer integrity

The formation of tight junctions (TJs) in ALI cultures was assessed for EBECs and ENECs in two ways. Firstly, the apical surface of each culture was assessed by eye for leakage of media; the presence of media on the apical surface of basolaterally fed cultures is indicative of TJ deterioration. Secondly, trans-epithelial electrical resistance (TEER) was measured between the time of seeding until the day of harvest, using an EVOM2 Voltohmmeter with STX-2 chopstick electrodes (World Precision Instruments, Stevenage, UK) immediately before the media exchange. For measurements, 0.5 mL and 1.5 mL of media were added to the apical and basolateral chambers, respectively, allowing the media to equilibrate to 37°C before measurements were performed in triplicate. All values were converted to Ohms/cm^2^ using Eq (1).


$$\text{Final}\;\text{TEER}\;\left(\Omega /{\text{cm}}^{2}\right)=\text{Net}\;\text{TEER}\;\left(\Omega \right)\times \text{Area}\;\text{of}\;\text{insert}\;\left({\text{cm}}^{2}\right)$$ (1)

### Ultra-thin section TEM

For transmission electron microscopy (TEM), samples were fixed in Karnovsky’s fixative (Polysciences, Inc., Warrington, PA) prepared to manufacturer’s recommendations for 1 h and washed with DPBS before post-fixation in 1% osmium tetroxide diluted in 0.1 M DPBS, dehydrated through serial ethanol solutions from 30 to 100% and placed in propylene oxide (Agar Scientific, Stansted, UK) prior to embedding in coffin-type moulds containing araldite resin (Agar Scientific). The resin was polymerised at 60°C for 48 h and semi-thin toluidine blue (Agar Scientific) stained sections (1 μm) prepared for light microscopy examination. Areas of membrane containing cellular material were selected for ultrastructural examination, with ultra-thin 90 nm thick sections cut using a diamond knife and prepared onto 100 μm copper grids, contrasted with uranyl acetate and lead citrate (Leica Biosystems GmbH, Wetzlar, Germany) prior to examination using an FEI TECNAI Bio-Twin 12 TEM (Thermo Fisher Scientific) equipped with a Deben AMT 4 K CCD camera (Deben, Bury St. Edmunds, UK) at 80 Kv.

### Gene expression assays

To perform relative quantification of gene expression, wells were aspirated of media and processed to extract total RNA using the RNeasy Micro Kit (Qiagen, Hilden, Germany). First strand cDNA synthesis was achieved using qScript cDNA supermix (Quantabio, Beverly, MA), performing the reaction according to manufacturer’s specifications. The following Taqman gene expression assays (FAM-MGB labelled) were obtained from Thermo Fisher Scientific: *MUC5AC* (Hs01365616_m1), *FOXJ1* (Ec07053643_g1), *NOTCH2* (Ec07043762_m1), *IFNB1* (Hs01077958_s1), *MUC5B* (Hs00861595_m1), *IL-6* (Ec03468678_m1), *IL-8* (Ec03468860_m1). These were normalised to the housekeeper gene, VIC-MGB labelled *GAPDH* (Ec03210916_gH). Assays targeted to *Homo sapiens* (Hs) sequences were listed as cross-reactive to *Equus caballus* (Ec). Real-time polymerase chain reaction was performed using the Brilliant III Ultra-fast QPCR Master Mix (Agilent Technologies, Santa Clara, CA), as per the manufacturer’s protocol, on a Bio-Rad T100 Thermal Cycler (Bio-Rad, Hercules, CA). Relative quantification for each target was calculated using duplicates of each assay pair, using the Livak method [57]. In the comparison of asthma biomarkers of asthma versus healthy controls, cycle threshold (Ct) values of target genes in asthmatic and healthy control groups were normalised to GAPDH, relative to equine total lung RNA (Amsbio, Abingdon, UK).

### Statistics

Analyses were performed using GraphPad Prism v9.0.0 for Windows (GraphPad Software, La Jolla, CA, www.graphpad.com). Replicate numbers of samples and experiments can be found as part of figure legends. For TEER analysis, an average of three readings per insert was performed at each time point, with n = 3 inserts per group, per experiment. TEER data represents three experiments performed on separate occasions and are presented as Mean ± SD, analysed using an Unpaired t-test. Differences in TEER measurements were analysed using a one-way ANOVA with a Tukey’s multiple comparisons test as follow-on analysis, presenting values as Mean ± Standard Deviation (SD), where n represents individual inserts. Statistical significance has been denoted as * p ≤ 0.05, ** p ≤ 0.01, *** p ≤ 0.001 and **** p ≤ 0.0001 (Tukey’s multiple comparisons test shown). Relative quantification of asthma mRNA biomarkers by real-time quantitative PCR of healthy versus asthmatic donors was analysed by multiple t-tests with Welch’s correction, significance denoted as above.
